# Description of 22 new alpha-1 antitrypsin genetic variants

**DOI:** 10.1186/s13023-018-0897-0

**Published:** 2018-09-17

**Authors:** Céline Renoux, Marie-Françoise Odou, Guillaume Tosato, Jordan Teoli, Norman Abbou, Christine Lombard, Farid Zerimech, Nicole Porchet, Colette Chapuis Cellier, Malika Balduyck, Philippe Joly

**Affiliations:** 1 Laboratoire de Biochimie et Biologie moléculaire Grand Est, UF “Biochimie des pathologies érythrocytaires”, Centre de Biologie et de Pathologie Est, Hospices Civils de Lyon, Lyon, France; 2 Laboratoire Interuniversitaire de Biologie de la Motricité (LIBM) EA7424, Team “Vascular Biology and Red Blood Cell”, Université Claude Bernard Lyon 1, Villeurbanne, France; 3 Service de Biochimie et Biologie moléculaire “Hormonologie, Métabolisme-Nutrition, Oncologie”, CHU Lille, F-59000 Lille, France; 4 Faculty of Pharmaceutical and Biological Sciences, UMR995, LIRIC (Lille Inflammation Research International Center), University of Lille, F-59000 Lille, France; 5 Laboratoire d’Immunologie, Centre Hospitalier Lyon-Sud, Hospices Civils de Lyon & Université Claude Bernard-Lyon 1, Lyon, France; 6 EA4483, IMPECS, Institut Pasteur de Lille, University of Lille, F-59000 Lille, France; 7 Faculty of Pharmaceutical and Biological Sciences, EA7364, RADEME (Research team on rare developmental and metabolic diseases), University of Lille, F-59000 Lille, France

**Keywords:** Alpha-1 antitrypsin deficiency, *SERPINA1* genotyping, Null alleles

## Abstract

Alpha-1 antitrypsin deficiency is an autosomal co-dominant disorder caused by mutations of the highly polymorphic *SERPINA1* gene. This genetic disorder still remains largely under-recognized and can be associated with lung and/or liver injury. The laboratory testing for this deficiency typically comprises serum alpha-1 antitrypsin quantification, phenotyping according to the isoelectric focusing pattern and genotyping if necessary. To date, more than 100 *SERPINA1* variants have been described and new genetic variants are frequently discovered. Over the past 10 years, 22 new genetic variants of the *SERPINA1* gene were identified in the daily practice of the University Medical laboratories of Lille and Lyon (France). Among these 22 variants, seven were Null alleles and one with a M1 migration pattern (M1_Cremeaux_) was considered as deficient according to the clinical and biological data and to the American College of Medical Genetics and Genomics (ACMG) criteria. Three other variants were classified as likely pathogenic, three as variants of uncertain significance while the remaining ones were assumed to be neutral. Moreover, we also identified in this study two recently described *SERPINA1* deficient variants: Trento (p.Glu99Val) and S_Donosti_ (p.Ser38Phe). The current data, together with a recent published meta-analysis, represent the most up-to-date list of *SERPINA1* variants available so far.

**Electronic supplementary material:**

The online version of this article (10.1186/s13023-018-0897-0) contains supplementary material, which is available to authorized users.

Alpha-1 antitrypsin (A1AT) is the main circulating protease inhibitor, protecting the lung parenchyma against proteolytic attacks. Alpha-1 antitrypsin deficiency (AATD) is a common but still largely under-recognized genetic disorder. It predisposes to liver and lung diseases and rarely to granulomatosis with polyangiitis and necrotizing panniculitis [1]. The wild-type allele is called PI*M while the most common deficient alleles are known as PI*S and PI*Z, according to their isoelectrofocusing (IEF) pattern. AATD-associated liver disease, observed for the deficient variants Z, S_Iiyama_ and M_Malton_, can be attributed to intracellular polymerization of the misfolded protein leading to endoplasmic reticulum storage disease. Mild liver storage is observed with the S variant which is probably degraded before secretion [2].

The medical indications for AATD screening were either a pulmonary or hepatic disorder or when a routine protein electrophoresis fortuitously revealed a splitting (with or without decrease) of the α_1_-globulin fraction at protein electrophoresis. The biochemistry laboratories of the academic medical centers of Lyon and Lille (France) currently investigate AATD by serum immunochemical quantification and IEF of A1AT. In the laboratory of Lyon, IEF is carried out on polyacrylamide gels based on the method previously described [3] with slight modifications of pH gradient (4.2–4.9). In the laboratory of Lille, IEF is performed on agarose gels using commercially available kits and immuno-enzymatic revelation (Sebia, Evry, France) [4]. In both laboratories, A1AT inhibitory activity may also be assessed through the serum elastase inhibitory capacity (SEIC) which relies on the inhibition measurement of the hydrolytic activity of the porcine pancreatic elastase by A1AT on a chromogenic substrate (N-Succinyl-Ala-Ala-Ala-p-nitroanilide). This kinetic spectrophotometric test, adapted from the method previously described by Klumpp and Bieth [5], was developed in close collaboration by the two laboratories so that the results could be comparable [6]. Using the correlation between A1AT concentration and SEIC, a theoretical SEIC can be calculated and compared to the measured SEIC with R being the ratio between the measured SEIC and the expected SEIC. For patients in heterozygosity with a new variant, R below 0.8 is presumptive of a dysfunctional variant.

This combination of techniques is sufficient to characterize up to 95% of A1AT abnormalities, mainly ZZ, SZ and SS phenotypes [1, 6, 7]. For the other cases (i.e. unexplained low A1AT level, unusual IEF pattern or IEF pattern inconsistent with clinical history), Sanger sequencing of the *SERPINA1* gene including coding exons, 5′ and 3′ untranslated regions (UTRs) and splice boundaries is performed and can be extended to intronic sequences by Next Generation Sequencing technology [8]. All sequence variations are named according to the Human Genome Variation Society (HGVS) and using the reference transcript NM_000295.4 which includes the 24 residues of the signal peptide.

Over the past 10 years, more than 1200 A1AT genotyping analyses performed in our two centers led to the identification of 22 new variants in 35 patients aged from 7 to 81 years (Table 1 and Fig. 1). It is noteworthy that 4 of them were already cited but neither named nor phenotypically or clinically described [9]. According to their IEF pattern and the birth place of the probands, we named them S_Roubaix_, W_Saint-Avre_, M1_Lille_ and M1_Lyon_. The criteria of the American College of Medical Genetics and Genomics (ACMG) were used to classify these 22 variants as benign, likely benign, of uncertain significance, likely pathogenic, or pathogenic [10]. Since we did not have the possibility to test them in expression vectors like HEK293T/17 or Hepa1–6 cells, the available clinical and biochemical data of A1AT were considered, as well as the results of two in silico pathogenicity predictors, shown to have a sensitivity of 0.75 for *SERPINA1* mutations [11]. The first one, namely SIFT for Sorting Intolerant From Tolerant, ranges from 0.00 to 1 and is mainly based on amino-acid conservation scores. A SIFT score between 0 and 0.05 is highly predicting of an affected protein function. The second one, namely PolyPhen-2 HVAR, proposes a prediction confidence score between 0.00 and 1.00 which uses multiple alignment and protein structural data. A PolyPhen-2 score higher than 0.8 is considered as probably damaging. The recently described REVEL (for Rare Exome Variant Ensemble Learner) method [12] was also used since it had been shown to be the most suitable one for the prediction of pathogenic A1AT variants [11]. Briefly, a REVEL score of less than 0.354 is highly predictive of a benign character of the variant whereas a score of more than 0.618 is highly predictive of pathogenicity.

**Table 1:** Molecular, biological and clinical characteristics of the 22 new *SERPINA1* variants

Variant name	NM_000295.4 (24 amino-acids signal peptide included)				Genetic back-ground	Clinical data				Biological data						ACMG score^c^
	dbSNP or Clinvar ID	Exon (II-V)	c.DNA	AA change		Sex	Age (years)	Circumstance of discovery	Pulmonary/hepatic status	AAT^a^ (g/L)	SEIC^a^ (IEU/L)	R^b^	IEF (PI)	CRP (mg/L)	Genotype	
G_Saint-sorlin_	/	Exon V	1252A > T	Lys418^*^	M2	♀	34	IgA nephropathy	No	2.06	37,164	1.28	GM3	19	G_Saint-sorlin_ M3	3
M1_Brest_	rs774775536	Exon IV	962A > G	Tyr321Cys	M1	♀	19	Familial screening	No	0.66	11,020	1.07	MZ	na	M1_Brest_ Z	2
M1_Bruxelles_	/	Exon II	116A > T	His39Leu	M1	♂	49	Elevated plasma GGT	Cholestasis	0.83	12,423	1.00	Heterogeneous pattern	na	M1_Bruxelles_ Z_Augsburg_	2
M1_Cremeaux_	/	Exon V	1074 T > A	His358Gln	M1	♀	39	Abnormal serum protein electrophoretic pattern	No	0.23	na	na	na	na	M1_Cremeaux_ Z	5
						♀	19	Familial screening	No	1.01	na	na	na	na	M1_Cremeaux_ M1	
						♀	37	Familial screening	No	0.88	11,120	0.83	na	na	M1_Cremeaux_ M2	
						♂	15	Familial screening	No	na	na	na	na	na	M1_Cremeaux_ M1	
M1_Lille_	rs141095970	Exon III	879C > A	His293Gln	M1	♀	33	Hepatic cytolysis Cholestasis, SLE	Cirrhosis	1.45	21,625	1.06	M	< 3	M1_Lille_ M1	2
M1_Lyon_	rs141620200	Exon IV	922G > T	Ala308Ser	M1	♀	10	Cystic fibrosis	Liver transplant	1.66	na	na	na	na	M1_Lyon_ Z	2
						♂	40	Familial screening	No	1.15	16,165	0.96	M1S	na	M1_Lyon_ S	
						♂	7	Familial screening	No	1.14	14,172	0.85	M1 M2	na	M1_Lyon_ M2	
						♂	15	Immune deficiency	No	1.38	19,240	0.99	M	na	M1_Lyon_ M1	
						♂	79	na	Emphysema	2.35	32,937	1.03	M	na	M1_Lyon_ M1	
						♀	79	na	Bronchiectasis	2.20	28,510	0.95	M	na	M1_Lyon_ M1	
						♂	36	Fertility tests	No	0.70	9556	0.88	MZ	na	M1_Lyon_ Z	
						♀	46	Familial screening	No	0.82	11,190	0.90	MZ	na	M1_Lyon_ Z	
M_Rouen_	rs764726147	Exon II	188G > A	Arg63His	M1/M2	♂	45	Familial screening	No	na	na	na	na	na	M_Rouen_ M1 or M_Rouen_ M2	3
M1_Saint-rambert_	/	Exon II	356G > T	Gly119Val	M1	♀	73	Solitary bone plasmocytoma	No	1.63	21,879	0.94	M1	17	M1_Saint-rambert_ M1	2
					M1	♀	37	na	No	na	na	na	ni	na	M1_Saint-rambert_ M2	
O_Thonon-les-bains_	rs759578830	Exon II	547G > A	Asp183Asn	M1	♀	43	Irritable Bowel syndrome	No	1.30	15,521	0.82	M2O	5	M2 O_Thonon-les-bains_	2
P_Loyettes_	rs766260108	Exon III	734 T > C	Met245Thr	M1	♀	71	CLL and type 2 diabetes	No	1.26	11,347	0.62	PS	23	P_Loyettes_ S	4
P_Solaize_	RCV000206568.1	Exon III	735G > A	Met245Ile	M2	♀	18	Crohn’s disease	No	1.26	14,318	0.79	M3Pfast	^**d**^	M3 P_Solaize_	4
S_Roubaix_	rs11575873	Exon II	211A > C	Ser71Arg	M1	♀	69	Cholestasis	HCV Cirrhosis	1.29	18,314	1.00	MS	60	M2 S_Roubaix_	2
W_Saint-Avre_	rs537285845	Exon II	436G > A	Glu146Lys	M1	♂	34	Abnormal serum protein electrophoretic pattern	No	0.82	9871	0.80	ni	na	W_Saint-Avre_ Z	3
					M1	♂	8	Biliary atresia	Pre-liver transplant data, probably on inflammatory status	1.47	na	na	M1W	na	M1 W_Saint-Avre_	
W_Vernaison_	/	Exon II	449 T > G	Leu150Arg	M1	♀	80	MALT lymphoma Sjogren’s syndrome Systemic necrotizing vasculitis	No	1.10	12,376	0.79	SW	35	S W_Vernaison_	4
X_Curis_	rs755851961	Exon III	811A > G	Asn271Asp	M1	♀	21	Cystic fibrosis	No	1.34	24,121	1.24	M2X	2	M2 X_Curis_	2
Q0_Achicourt_	rs750779440	Intron 3	917 + 1G > A	/	S	♂	59	Dyspnea	Emphysema	< 0.10	2045	ns	No band	< 3	Q0_Achicourt_ Q0_Clayton_	5
Q0_Amiens_	rs781591420	Intron 4	1065 + 1G > A	/	M1	♀	81	Abnormal serum protein electrophoretic pattern	No	1.18	17,419	1.03	M	na	M1 Q0_Amiens_	5
						♀	35	Neutropenia	No	0.76	11,741	1.01	M	< 3	M3 Q0_Amiens_	
Q0_Casablanca_	RCV000408906.1	Exon II	288_291del	His97Metfs^*^7	M2	♂	21	Neutropenia	Bronchiectasis	< 0.10	3747	ns	No band	15	Q0_Casablanca_ homozygous	5
Q0_Lille_					Z	♂	36	Pneumothorax	Recurrent pneumothorax	1.40	19,317	0.98	M	231	M1 Q0_Lille_	5
Q0_Montluel_	rs760849035	Exon V	1237_1239del	Val413^*^	M1	♀	51	Thrombophilia screening	No	0.66	7547	0.72	M1	5	M1 Q0_Montluel_	5
Q0_Saint-Avold_	/	Intron 3	918 – 1G > A	/	M1	♀	63	na	Emphysema	0.21	5898	1.30	Z	na	Q0_Saint-Avold_ Z	5
Q0_Saint-Etienne_	/	Exon II	559A > T	Lys187^*^	M4	♂	25	AATD familial screening	No	0.74	6647	0.58	M3	na	M3 Q0_Saint-Etienne_	5

CRP: C-Reactive Protein

*na* not available, *ni* not interpretable (unusual IEF pattern), *ns* not significant, *CLL* chronic lymphocytic leukemia, *GGT* gamma-glutamyl transpeptidase, *HCV* hepatitis C virus, *MALT* mucosa-associated lymphoid tissue, *SLE* Systemic lupus erythematosus

^a^ Normal ranges in serum: A1AT: 0.90–2.00 g/L; SEIC (serum elastase inhibitory capacity): 17,500–31,500 IU/L.

^b^ R = measured SEIC / expected SEIC; expected SEIC is based on the correlation between the measured SEIC and the corresponding AAT level according to the following linear relationship established from 10,863 individuals: SEIC (IU/L) = 12,784 x A1AT (g/L) + 1855. Measured SEIC< 17,500 IEU/L and/or *R* < 0.8 may result from A1AT functional deficiency

^c^ ACMG classification: 1 = benign, 2 = likely benign, 3 = uncertain significance, 4 = likely pathogenic, 5 = pathogenic

^d^ inflammatory electrophoretic profile

*nomenclatura rule for stop codon

**Fig. 1:**
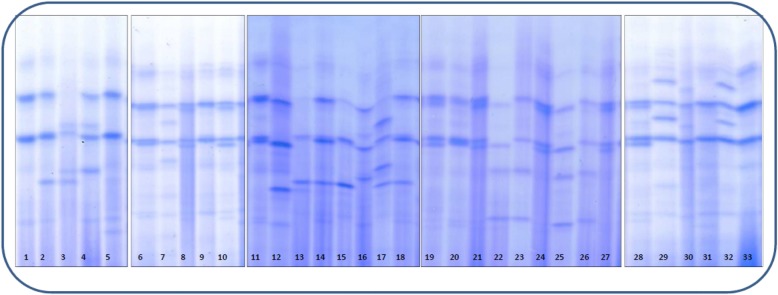
IEF patterns of some frequent and rare A1AT phenotypes (polyacrylamide gels with Coomassie blue staining). 1, 33: M_1_M_3;_ 2, 15, 18: M_1_S_;_ 3, 17: P_Loyettes_ S; 4: M_3_P_Loyettes;_ 5, 31: M_1_Z; 6, 11, 20: M_1_M_4;_ 7: M_3_P_Solaize;_ 8, 10, 19, 21, 24, 27, 28: M_1_M_2;_ 9:M_1_M_1_ 12: M_2_S_Roubaix;_ 13: SW_Vernaison;_ 14: M_3_S; 16: M_2_P; 22: M_2_X_Christchurch;_ 23: M_1_X_Christchurch;_ 25: M_2_X_Curis;_ 26: M_1_X_Christchurch;_ 29, 32: G_Saint-Sorlin_M_1;_ 30: IM_3_

Seven new variants were assumed to be Null ones: Q0_Lille_, Q0_Casablanca_, Q0_Saint-Etienne_, Q0_Achicourt_, Q0_Saint-Avold_, Q0_Amiens_ and Q0_Montluel_. They resulted from splice-site, non-sense or frame shift mutations leading to premature stop codons with biosynthesis of truncated proteins or pre-mRNA degradation by the nonsense mediated decay mechanism. Interestingly, the c.288_291del frame shift mutation gives rise to two different *SERPINA1* Null variants which are associated with distinct genetic backgrounds: M2 for Q0_Casablanca_ and Z for Q0_Lille_. The c.559A > T (Q0_Saint-Etienne_) and c.1237_1239del (Q0_Montluel_) mutations lead to a premature stop codon while Q0_Achicourt_, Q0_Saint-Avold_ and Q0_Amiens_ are caused by splicing abnormalities. It is noteworthy that Q0_Achicourt_ and Q0_Saint-Avold_, found in young patients presenting with emphysema, were both in compound heterozygosity with another deficient *SERPINA1* allele (Q0_Clayton_ and Z, respectively).

The M1_Cremeaux_ variant was identified in four members of a same family (two sisters and their sons). The propositus was a 36-year-old woman without any pulmonary or hepatic disorder harboring the M1_Cremeaux_ variant in heterozygosity with the dysfunctional Z variant. A1AT biochemical analysis was prescribed because of low α_1_-globulin fraction at protein electrophoresis during a hair loss exploration. Despite the absence of any specific clinical impact, M1_Cremeaux_ was considered as a deficient A1AT variant (ACMG class5) for four reasons: (i) the A1AT serum level was significantly decreased (0.23 g/L in heterozygosity with the Z allele and from 0.88 to 1.01 g/L in association with a M1 or M2 allele), (ii) the mutation was located at the beginning of the 5Aβ-strand which is an important region for the protein stability [1] (iii) the pathogenic A1AT King variant affects the same amino-acid (p.His358Asp) [13] and (iv) the SIFT score (0.48) was normal but the PolyPhen-2 and REVEL scores (0.999 and 0.650) were highly predictive of pathogenicity.

The two P variants, P_Loyettes_ and P_Solaize_, were suspected to be dysfunctional according to their decreased elastase inhibitory activity demonstrated by R values of 0.62 and 0.79, respectively. Sustaining our hypothesis, REVEL, SIFT and PolyPhen-2 scores predicted P_Loyettes_ (0.933, 0 and 1.00, respectively) and P_Solaize_ (0.597, 0 and 0.623, respectively) as deleterious. The W_vernaison_ variant also harbored a decreased elastase inhibitory activity (R value 0.79) and an IEF pattern with almost undetectable bands; nevertheless, SIFT and PolyPhen-2 scores predicted it as benign (0.08 and 0.432 respectively) but not the REVEL score of 0.638. Moreover, these three variants were identified in patients with an inflammatory status (CRP plasma levels higher than 10 mg/L) that probably led to overestimation of the recorded A1AT levels. They were thus classified as likely pathogenic according to ACMG criteria (class 4).

While caused by a non-sense mutation, A1AT G_Saint-Sorlin_ (c.1252A > T; p.Lys418*) was ranged as variant of uncertain significance (class 3) since the A1AT biochemical data were normal. As the premature stop codon is located on the very last triplet of the gene, the final protein lacks only one amino-acid and it seems to have no consequence on its synthesis or functional activity. Conversely, the M1_Rouen_ variant was also ranged in class 3 and not considered as benign or likely benign because: (i) it appears at very low allelic frequencies in databases (ExAC and Topmed: 0.0012%), (ii) a pathogenic variant on the same amino-acid (namely, the I variant p.Arg63Cys) has been described and (iii) we could not get any serum sample to assess A1AT quantification and SEIC. In detail, the SIFT and PolyPhen-2 algorithms classify the I variant as deleterious (0 and 1, respectively) while they are contradictory for the M1_Rouen_ variant (0.04 and 0.185, respectively). A border-line R ratio of 0.8 was obtained for an asymptomatic 34 -year -old woman harboring the W_Saint -Avre_ variant in heterozygosity with the dysfunctional Z variant. According to its low frequency in databases (ExAC: 0.0032%) and to its SIFT and PolyPhen-2 scores (1 and 0.000 respectively), W_Saint -Avre_ was also ranged in class 3 of ACMG classification.

The remaining eight variants were classified as likely benign (class 2) because in silico algorithms predicted no impact on gene product and the A1AT quantitation and SEIC measures revealed no abnormality.

Very interestingly, we also identified during the course of this study two *SERPINA1* deficient variants that were very recently described: Trento (p.Glu99Val) [14] and S_Donosti_ (p.Ser38Phe) [15]. The Trento variant showed compromised conformational stability after secretion from the hepatocyte [14]. In our cohort, this variant was present in heterozygosity with the M_Malton_ variant in a 42-year-old man with a low A1AT level (0.85 g/L) presenting with hepatic fibrosis. The S_Donosti_ variant was shown to form intra-cellular polymers that prevent its secretion from the hepatocytes. We identified the S_Donosti_ variant in two unrelated individuals (in heterozygosity with the M1 variant and with the S variant, respectively): (i) a 64-year-old woman suffering from emphysema (A1AT level = 1.21 g/L but inflammatory status not known) and (ii) a 41-year-old man suffering from hemochromatosis (A1AT level = 0.80 g/L).

In conclusion, this study highlights the importance of the whole *SERPINA1* gene sequencing (and not only the specific research of the Z and S variants) to explain some AATD clinical and biological pictures. Among these 22 new A1AT variants, a significant percentage of severely deficient ones (class 5) was observed (36.4%): Seven Q0 alleles and one deficient M1 allele (M1_Cremeaux_). Three variants (P_Loyettes_, P_Solaize_ and W_Vernaison_) could be classified as dysfunctional variants (class 4) mainly because of their reduced elastase inhibitory activity. Three variants (M1_Rouen_, G_Saint -Sorlin_ and W_Saint -Avre_) were classified as variants of uncertain significance (Class 3) and the eight remaining ones as likely benign (Class 2). To note, we fortuitously observed that the IEF pattern of the S_Roubaix_ variant depended on the migration medium: W-like on polyacrylamide gels (Lyon) and S-like on agarose gels (Lille) (Additional file 1: Figure S1). Since all patients carrying the S_Roubaix_ variant were of North African origin, we highly speculate that this variant might correspond to the ‘old’ W3_Constantine_ described in 1977 by Khitri [16]. The recent meta-analysis by Silva et al., completed by the present data, represents the most up-to-date list of *SERPINA1* variants available so far.

## Additional file

Additional file 1: **Figure S1.** A1AT phenotypes: (A) Coomassie blue stained polyacrylamide gel (B) agarose gel followed by immunofixation. 1:M_1_; 2:M_2_S; 3:M_1_M_4_; 4,5,6: M_2_P; 7:M_2_S_Roubaix_; 8: IM; 9:M_2_S_Roubaix_; 10,11:M_1_Z; 12:M_1_; 13:M_2_S; 14:M_1_S. The S_Roubaix_ variant has clearly different patterns of migration on polyacrylamide and on agarose gels. (PPTX 217 kb)

## Abbreviations

A1AT: Alpha-1-antitrypsin
AATD: Alpha-1-antitrypsin deficiency
IEF: Isoelectric focusing
SEIC: Serum elastase inhibitory capacity
