# Supplementary figures and images for: Description of 22 new alpha-1 antitrypsin genetic variants
Source: Orphanet J Rare Dis. 2018 Sep 17;13:161. doi: 10.1186/s13023-018-0897-0 (PMC6142351; doi:10.1186/s13023-018-0897-0)

## Slide 1
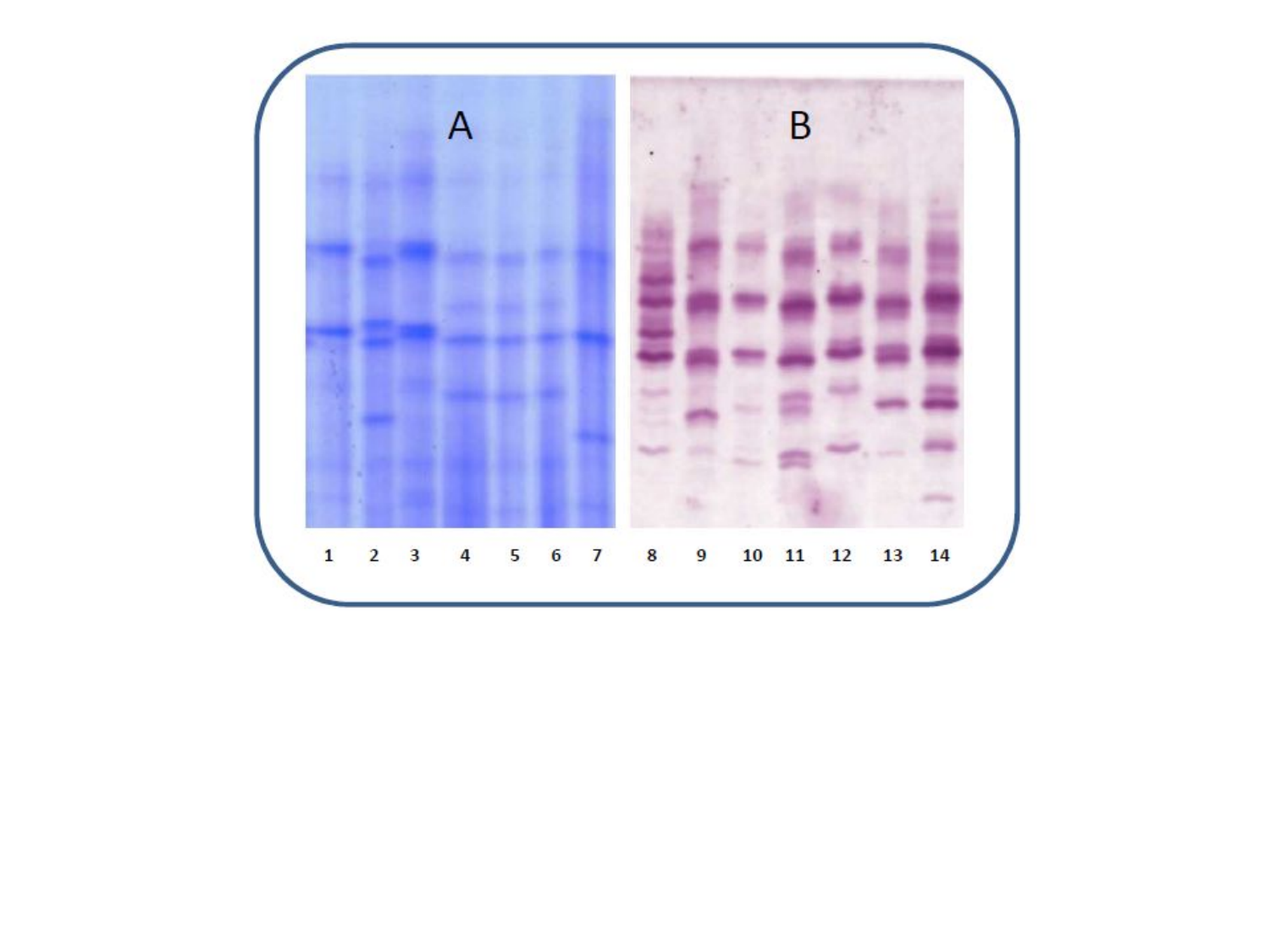

Supplement: Supplementary file 1 — Figure S1. A1AT phenotypes: (A) Coomassie blue stained polyacrylamide gel (B) agarose gel followed by immunofixation. 1:M1; 2:M2S; 3:M1M4; 4,5,6: M2P; 7:M2SRoubaix; 8: IM; 9:M2SRoubaix; 10,11:M1Z; 12:M1; 13:M2S; 14:M1S. The SRoubaix variant has clearly different patterns of migration on polyacrylamide and on agarose gels. (PPTX 217 kb) [file 13023_2018_897_MOESM1_ESM.pptx]
